# Crambescidin-816 Acts as a Fungicidal with More Potency than Crambescidin-800 and -830, Inducing Cell Cycle Arrest, Increased Cell Size and Apoptosis in *Saccharomyces cerevisiae*

**DOI:** 10.3390/md11114419

**Published:** 2013-11-08

**Authors:** Juan A. Rubiolo, Eva Ternon, Henar López-Alonso, Olivier P. Thomas, Félix V. Vega, Mercedes R. Vieytes, Luis M. Botana

**Affiliations:** 1 Department of Pharmacology, Faculty of Veterinary, University of Santiago de Compostela (USC), Campus Lugo, Lugo 27002, Spain; E-Mails: ja.rubiolo@usc.es (J.A.R.); henar.lopez@usc.es (H.L.-A.); 2 Department of Physiology, Faculty of Veterinary, University of Santiago de Compostela (USC), Campus Lugo, Lugo 27002, Spain; E-Mails: felixvictor.vega@usc.es (F.V.V.); mmercedes.rodriguez@usc.es (M.R.V.); 3 Nice Institute of Chemistry-PCRE, UMR 7272 CNRS, Faculty of Sciences, University of Nice Sophia Antipolis, Parc Valrose 06108 Nice, France; E-Mails: eva.ternon@unice.fr (E.T.); olivier.thomas@unice.fr (O.P.T.)

**Keywords:** crambescidine-816, antifungal, cell cycle arrest, apoptosis

## Abstract

In this paper, we show the effect of crambescidin-816, -800, and -830 on *Saccharomyces cerevisiae* viability. We determined that, of the three molecules tested, crambescidin-816 was the most potent. Based on this result, we continued by determining the effect of crambescidin-816 on the cell cycle of this yeast. The compound induced cell cycle arrest in G2/M followed by an increase in cell DNA content and size. When the type of cell death was analyzed, we observed that crambescidin-816 induced apoptosis. The antifungal effect indicates that crambescidins, and mostly crambescidin-816, could serve as a lead compound to fight fungal infections.

## 1. Introduction

Marine sponges are among the most important producers of secondary metabolites with antimicrobial effect. After centuries of evolutionary pressure, these sessile marine filter feeders have developed a very efficient chemical arsenal against virus, bacteria, and eukaryotic organisms. More than 200 new metabolites from this group of animals are reported each year and more than 5300 different secondary metabolites are known from sponges [1,2]. Meanwhile, in the last decade, many compounds with antifungal activity have been isolated from sponges (for comprehensive reviews see [3,4,5,6]) indicating that this group of organisms is a valuable source of this type of compounds. The sponge *Crambe crambe* commonly found in the rocky costs of the Mediterranean sea, is capable of inducing necrosis of other sponge tissues when they are kept in contact [7], and was initially reported to produce potent antibacterial and antifungal compounds [8]. Additionally, metabolites of this highly toxic sponge have proven to have antifouling activity against microfoulers and invertebrate larvae [9]. Two families of guanidine alkaloids have been isolated from *Crambe crambe*, namely crambescidins and crambescins. Some of these have been shown to have mid nanomolar cytotoxicities against human tumor derived cell lines [10]. Crambescidin-800 (C800) induces differentiation of K562 leukemia cells leading to cell cycle arrest in S-phase [11]. C800 was initially identified as a promising antiviral and antifungal compound [10], and later purified from *Monanchora unguifera* in the search of antifungal leads, after a high-throughput screening with the aim of discovering biologically active small molecules, using *Saccharomyces cerevisiae* as model organism. This compound presented an IC_20_ in liquid culture of 0.83 µM, similar to that of the most active selected compounds from the NCI diversity set (collection of 2000 synthetic small molecules selected from the full NCI screening collection) [12]. Another crambescidin isolated from *Crambe crambe* is crambescidin-816 (C816). This compound has been shown to exert a Ca^2+^ antagonistic activity with higher potency than nifedipine [13,14]. It is also active against human colon carcinoma cells [13].

**Figure 1:**
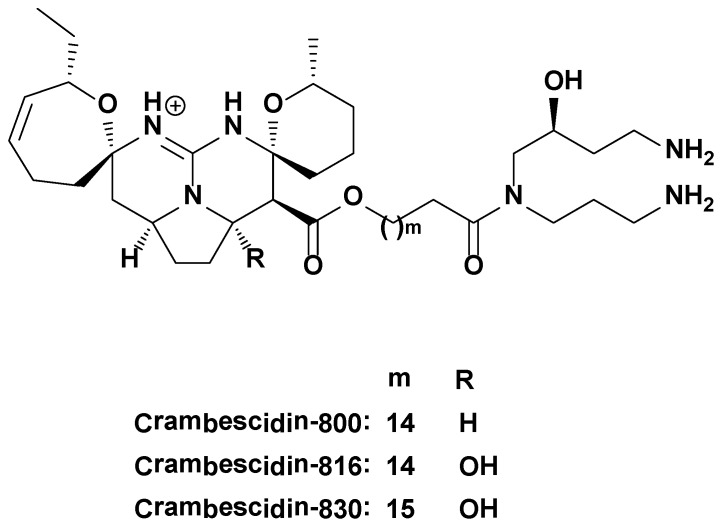
Chemical structures of crambescidin-816, -800, and -830.

Due to the problems associated with current antifungal drugs, and the increasing number of invasive fungal infections in immunocompromised patients, a new generation of antifungal drugs is urgently needed. Since crambescidin-800 has shown promising antifungal activity, and based on the data available for crambescidin-816 showing that it has a higher activity than crambescidin-800 in eukaryotic cells, we tested the antifungal effect of crambescidin-816 in comparison with crambescidin-800 and another of its family members -830 (C830, Figure 1) in *S. cerevisiae,* which is recognized to be a good model for antifungal drug development [15]. We herein show that crambescidin-816 reduces cell viability in *S. cerevisiae* inducing an increment in cell size and DNA content, and apoptosis. The potency of this compound is 10 times higher than that of crambescidin-800 and 4 times higher than that of crambescidin-830.

## 2. Results and Discussion

### 2.1. C816 Is More Potent on *S. cerevisiae* than C800 and C830, and Has a Fungicidal Effect

**Figure 2:**
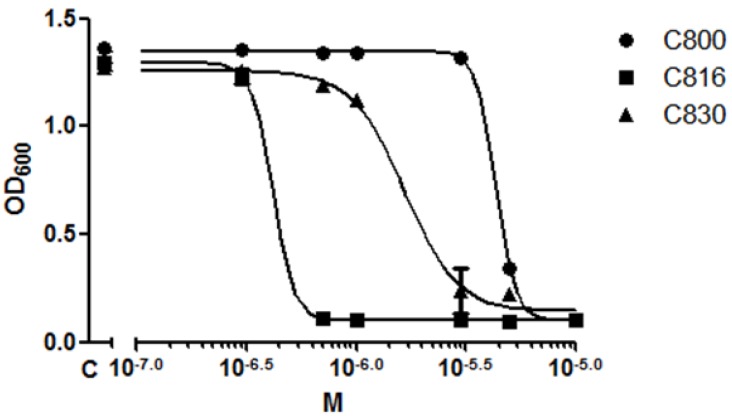
Determination of the MIC_50_ for C800, C816 and C830 in *S. cerevisiae* after 24 h. The optical density determined for each treatment was plotted against the concentrations tested, expressed in molarity. The mean (*n* = 4) ± SD for each treatment is shown.

We compared the effect of C816, C800 and C830 on *S. cerevisiae* cell growth in order to determine their relative potencies. We observed that the most potent molecule was C816 followed by C830 and C800 with MIC_50_ (the antimicrobial concentration the inhibits 50% of the growth) at 24 h of 0.557 (95% confidence interval = 0.5116 to 0.608), 1.650 (95% confidence interval = 1.374 to 2.011), and 4.316 (95% confidence interval = 4.210 to 4.427) µM respectively. C816 was almost 10 times more potent than C800 (Figure 2).

As previously stated in the introduction, C800 has been previously identified as a potent antifungal agent with an IC_20_ of 0.83 µM [12]. At the concentration reported by these authors we observed no effect on *S. cerevisiae* cell growth after 24 h. This could be due to the different approaches used for the determination of the effect of the tested compound on cell growth. As shown below, C816 induces a delay of cell growth after short time periods, which is not observed after 24 h. This could explain why the cited authors observed an inhibition of 20% of cell growth at a concentration of 0.83 µM while we determined a minimum inhibitory concentration of 4.316 µM after 24 h. An increased lag phase, induced by C800 could produce an IC_20_ (defined as the concentration in which the doubling time of the DMSO control divided by the doubling time with compound was equal to 0.8) lower than that observed after incubation for longer time periods. Also, a difference resulting as a consequence of using different strains of *S. cerevisiae* cannot be ruled out. We continued our study with the most potent compound, C816.

**Figure 3:**
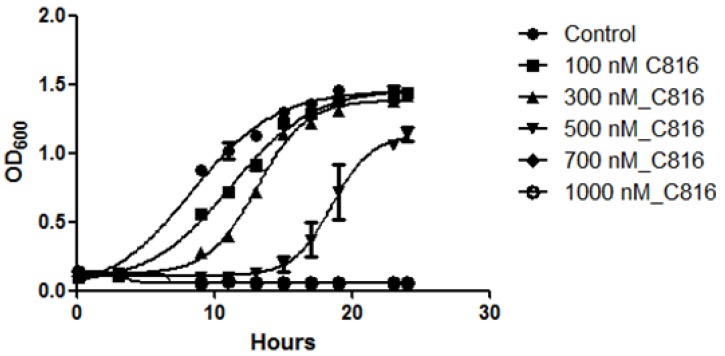
Growth inhibition of *S. cerevisiae* cultures treated with various doses of C816 (100–1000 nM). No growth was observed in cultures treated with 700 and 1000 nM C816. The mean (*n* = 4) ± SD for each treatment is shown.

To investigate the effect of C816 on *S. cerevisiae* growth, cultures of this organism were exposed to C816 up to 24 h, and cell growth was monitored measuring the OD of the cultures. We determined that the tested compound inhibited *S. cerevisiae* growth in a dose dependent manner (Figure 3). At the two highest concentrations tested no cell growth was observed up to 24 h. Lower concentrations (100 and 300 nM) were capable to delay the growth of the culture after the first 15 h but finally the cultures reached the same density as control cells after 24 h.

**Figure 4:**
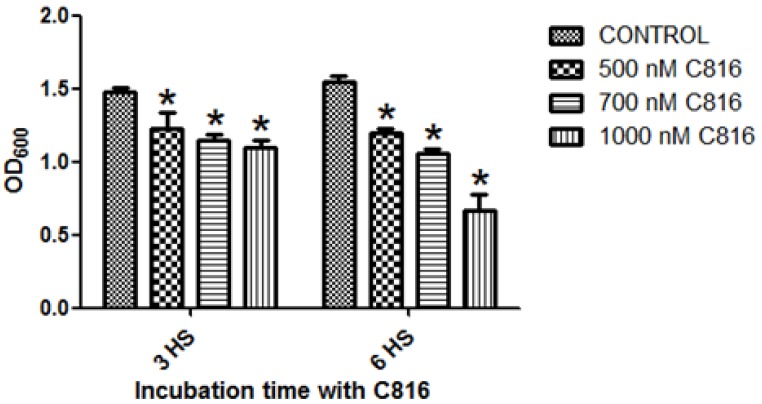
Determination of the fungicidal effects of C816 on *S cerevisiae*. Cultures were treated with 500, 700 and 1000 nM C816 for 3 or 6 h. After toxin treatment cells were washed and kept in culture for another 24 h. Culture growth was determined measuring the OD at 600 nm. Significant differences respect to control * *p* < 0.01, *n* = 4.

We then assayed if C816 was a fungistatic or if it acted as a fungicide in *S. cerevisiae*. Cells incubated with 300, 700 and 1000 nM C816 for 3 or 6 h and then cultured in toxin free medium up to 24 h (see Experimental Section for a detailed explanation of culture conditions) presented a reduced viability at both times tested (Figure 4). Cells exposed to C816 for 6 h showed a reduced growth when compared to those treated with the compound for 3 h. In both cases, a dose dependent response was observed.

The results show that C816 is cytotoxic for *S. cerevisiae*. After 24 h the cultures treated with the highest concentration tested, reached approximately half of the growth seen in controls. These results indicate that C816 acts as a fungicidal and not as a fungistatic.

### 2.2. C816 Induces Cell Cycle Arrest and an Increase in *S. cerevisiae* DNA Content

**Figure 5:**
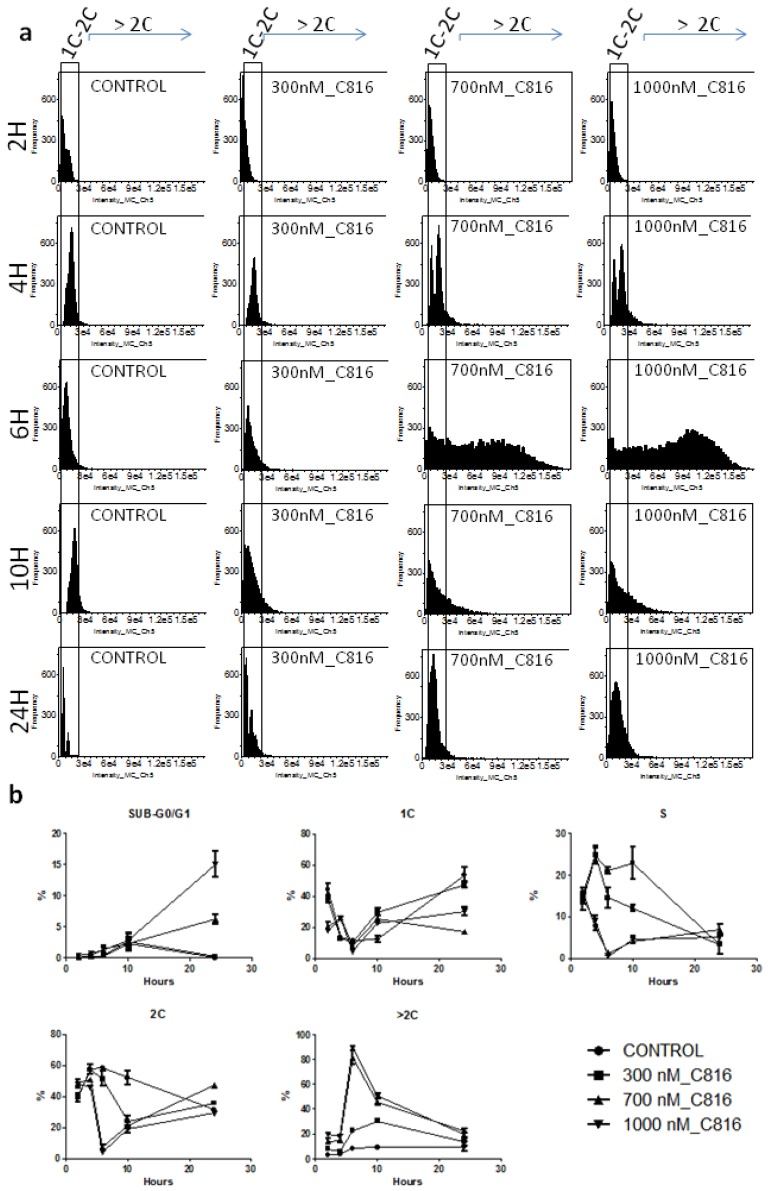
C816 induces an increase in the DNA content in *S.* cerevisiae. (**a**) Histograms obtained after flow cytometry of *S. cerevisiae* treated with C816 for 2, 4, 6, 10 and 24 h. Marked with a rectangle are the cells with 1C, 2C cell cycle distribution. (**b**) Analysis of cell population distributions after the different treatments performed. Each treatment was assayed in triplicate, and two experiments were performed. The mean (*n* = 3) ± SD for each treatment is shown.

To assay if C816 affected the cell cycle of *S. cerevisiae,* cultures of these cells were treated with C816 and then studied by imaging flow cytometry. Examples of histograms obtained are shown in Figure 5a. We observed what initially appeared as cell cycle arrest in G2/M in the first 2 h of culture as determined by the increased population of cells in this phase of the cell cycle for cultures treated with 700 and 1000 nM C816. Longer incubation periods indicated that C816 induced the appearance of cells with a broad range of DNA content. We observed a population of cells with ploidy higher than 4C, which was higher after 6 h of incubation with 700 and 1000 nM C816 and after 10 h of incubation with 300 nM C816 (Figure 5a). No difference was observed after 24 h. At this time point, an increase in cells with a DNA content below of 1C was detected, which is indicative of apoptosis (Figure 5b). Cells with up to 32C were detected at the two highest concentrations tested.

The increased DNA content in C816 treated cells could be due to increased mitochondrial DNA content [16]. A similar effect has been observed in *S. cerevisiae* after treatment with the microtubule-depolymerizing drug nocodazole, a compound that induced mitotic cell death with apoptotic-like features [17]. On the other hand, cell death could be a consequence of an abnormal morphogenetic response in the presence of C816 making the cells commit new rounds of DNA replication in the absence of cytokinesis, or cell cycle arrest in anaphase. It has been previously shown that *S. cerevisiae* can replicate their DNA in the absence of cytokinesis [18], as consequence of septa mislocalization [19,20]. This raises the possibility that crambescidins could interfere with cell wall synthesis or organization. The cortical actin cytoskeleton is essential for the development of a functional cell wall and is localized at particular areas at different stages of the cell cycle [21,22]. We have observed that C816 interferes with tumor cell actin cytoskeleton [23], so an effect of this compound on yeast cytoskeleton is also a possibility. Further research is needed to confirm these assumptions.

### 2.3. C816 Induces an Increase in Cell Size

**Figure 6:**
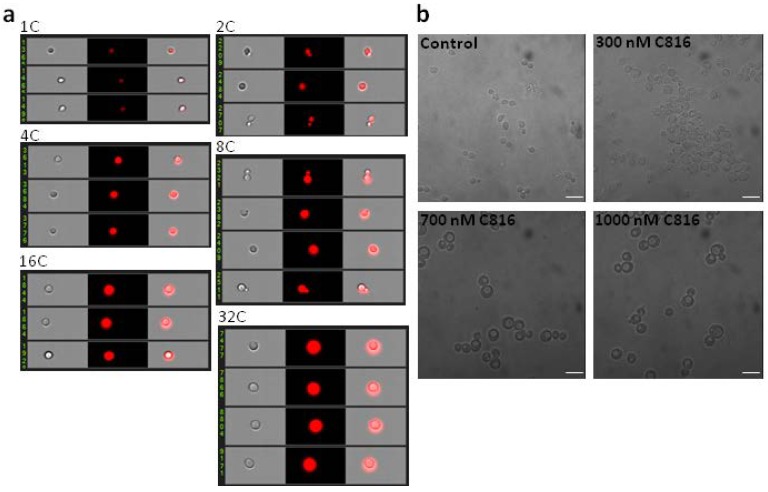
C816 induces an increment of the cell size in *S. cerevisiae*. (**a**) Representative images of cells in the different populations of the cell cycle after treatment with C816 obtained by imaging flow cytometry. Control samples showed cell with 1 and 2C, while C816 treatment induced the appearance of cells with 4, 8, 16 and 32C. (**b**) Bright field microscopy of control and C816 treated *S. cerevisiae*. An increase in cell size can be observed in treated cells, especially in cells treated with 700 and 1000 nM C816. Scale bar = 10 µm.

The images obtained after imaging flow cytometry showed, besides an increase in DNA content, an increment in cell size that appeared early after treatment (Figure 6a). This was also observed after inspection of the cultures by bright field microscopy (Figure 6b).

An increase of cell size is expected since the higher DNA content requires an augmented cell size to accommodate the genetic material.

### 2.4. C816 Induces Apoptosis in *S. cerevisiae*

In cdc13-1 cells (*S. cerevisiae* mutant for CDC13, a telomere binding protein that prevent degradation of telomeres) grown at restrictive temperature, it has been shown that increased cell size can result in cell lysis. These cells maintain high viability for several hours during which time continue to grow increasing two- to three-fold in diameter. When such cells reach a critical size, they lyse [24]. To assay if the increment in cell size induced by C816 could produce cell lysis, exponentially growing cultures of *S. cerevisiae* were treated with 300, 700, and 1000 nM C816. After 10 h the cells were washed and then incubated in the presence of PI to determine the integrity of the plasma membrane. Treated cells did not show an increased presence of stained cells when compared to controls (Supplementary Figure S1).

**Figure 7:**
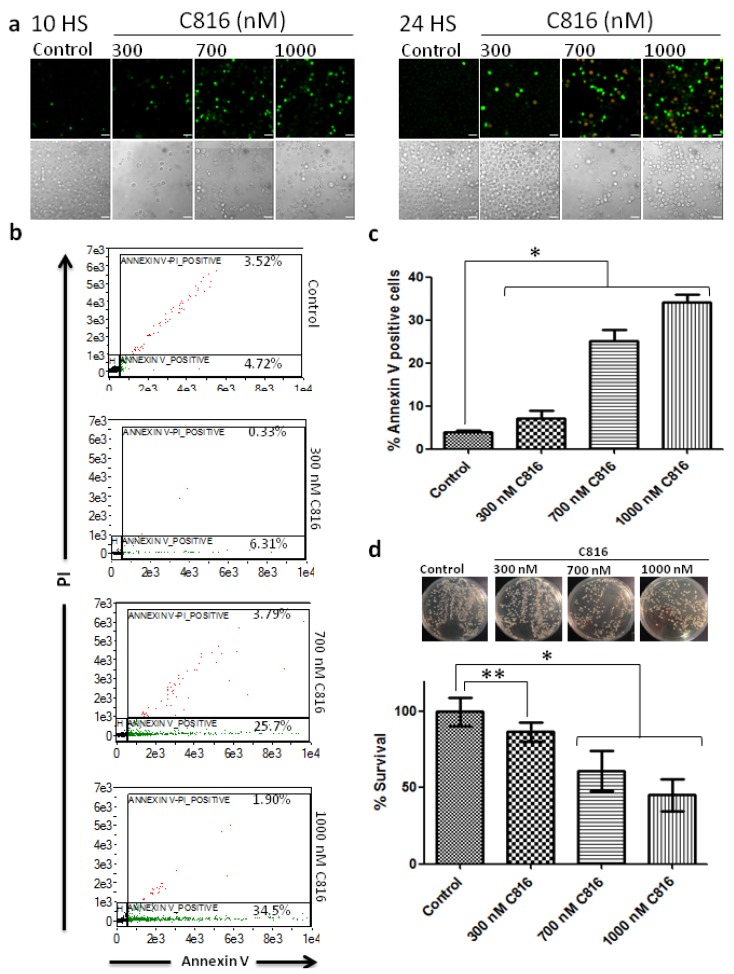
(**a**) Annexin V and PI staining of *S. cerevisiae* after C816 treatment for 10 and 24 h. Green: Annexin V. Red: PI. After 10 h only Annexin V staining is observed. PI staining is observed but after 24 h of treatment. Scale bar = 10 µm (**b**) Apoptosis determination by flow cytometry in *S. cerevisiae* after C816 treatment for 10 h. The toxin induced a dose dependent increase in Annexin V stained cells. Representative results are shown. (**c**) Quantification of Annexin V positive cells detected by flow cytometry. * Significant differences respect to control *p* < 0.01, *n* = 3. (**d**) Survival plating assay for cells treated as those analyzed by flow cytometry. After C816 treatment for 10 h, cells were serially diluted, plated and incubated at 30 °C. Colonies were counted after 48 h. * Significant differences respect to control *p* < 0.01, *n* = 3, ** significant differences respect to control *p* < 0.05, *n* = 3.

After discarding the existence of necrosis and, since yeast has been shown to undergo apoptosis by numerous types of insults [25], we analyzed if C816 induced apoptosis. As shown in Figure 5b, an increase in the sub-G0/G1 cell population was observed after 24 h of C816 treatment (see Supplementary Figure S2 for scaled up histograms that better show this population). To better assay if C816 induced apoptosis in *S. cerevisiae* we performed Annexin V and PI staining experiments. Annexin V has specific affinity to phosphatidylserine (PS). Soon after apoptosis is induced, PS translocates from the inner to the outer leaflet of the plasma membrane [26]. After translocation PS can readily bind Annexin V, which coupled to FITC, was detected by flow cytometry and fluorescence microscopy. PI was also used for apoptosis determination. It binds to DNA and is impermeant to live and apoptotic cells, but stains necrotic cells. Annexin V staining showed that C816 induces apoptosis in *S. cerevisiae*. As determined by fluorescence microscopy, after 10 h there was an important increase of Annexin V positive cells without PI staining, indicative of cells in early apoptosis, while after 24 h we observed Annexin V staining and the appearance of PI positive cells indicative of cells in late apoptosis (secondary necrotic) (Figure 7a). When cells were analyzed for Annexin V and PI staining by flow cytometry, we observed mainly a dose dependent increase in Annexin V stained cells after 10 h of C816 treatment (Figure 7b,c). At this time, a dose dependent reduction in cell survival was detected (Figure 7d). Supplementary Figure S3 shows representative images for healthy, Annexin V stained cells, and Annexin V-PI stained cells. As previously stated, apoptotic-like cell death has been previously described in *S. cerevisiae* after treatment with nocodazole [17]. Also, several other antineoplastic and antifungal drugs, some of which are actually used in the clinic like Amphotericin B, have already been demonstrated to induce apoptosis in yeast [27].

After apoptosis determination by fluorescence microscopy and flow cytometry, similar results were obtained showing an increase in the apoptotic population after 10 h. After a longer incubation period, PI staining was observed indicative of secondary necrosis. In unicellular eukaryotes, the apoptosis outcome is secondary necrosis were cytoplasmic membrane permeabilization occurs and PI staining is detected. This type of necrosis was named as being secondary to distinguish this mode of cell elimination from cellular necrosis occurring without apoptosis, termed primary necrosis [28].

### 2.5. C816 Disrupts the Mitochondrial Membrane Potential (ΔΨ_m_) in *S. cerevisiae*

**Figure 8:**
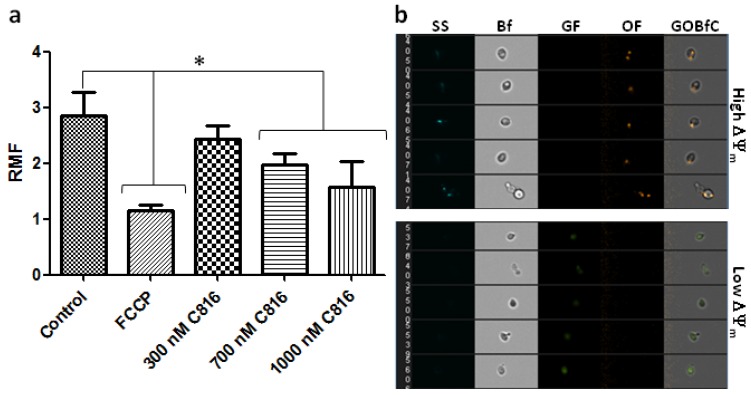
(**a**) Quantification of the relative mitochondrial fluorescence (RMF) in C816 treated *S. cerevisiae*. * Significant differences respect to control *p* < 0.01, *n* = 3. (**b**) Images obtained by imaging flow cytometry of cells with functional and damaged mitochondria. SS: Side scatter, Bf: Bright field, GF: Green fluorescence, OF: Orange fluorescence, and GOBfC: Composite of the bright field, green and red fluorescence.

Mitochondrial permeability transition is a critical step in the cellular apoptotic pathway [29], it precedes phosphatidylserine flip-flop and coincides with caspase activation [30]. This process produces the disruption of the electrochemical gradient in the mitochondria by pore formation [31]. The basic molecular machinery of programmed cell death has been shown to be phylogenetically conserved in yeast as well as animals [25]. Several compounds, like acetic acid [32,33], H_2_O_2_ [34], and aspirin [35] have been shown to induce apoptosis, involving mitochondrial dysfunction, in yeast. To determine if C816 affected the mitochondrial ΔΨ_m_, we treated *S. cerevisiae* with this compound for 6 h, the incubation period at which the highest ploidy was detected after exposure to 700 and 1000 nM C816, and then analyzed the mitochondrial function using the JC-1. This dye, in the presence of high mitochondrial ΔΨ_m_, forms aggregates with orange fluorescence. In cells with damaged mitochondria JC-1 remains in the monomeric form, which shows only green fluorescence [36]. After analysis by imaging flow cytometry the results showed that C816 reduced the mitochondrial ΔΨ_m_ at the two highest concentrations tested (Figure 8a). Even though 300 nM C816 appeared to decrease the mitochondrial ΔΨ_m_, the difference was not statistically significant. Examples of cells with healthy and damaged mitochondria detected after imaging flow cytometry are shown in Figure 8b.

Increase in DNA content and mitochondrial depolarization with the consequent apoptosis could be related since hyperploid cells, which show an increased size, also show loss of mitochondrial membrane potential. We are currently trying to determine if this is the case.

*Saccharomyces cerevisiae*, which is closely related to fungi harmful for humans [37], has been shown to be a good model system for studying the properties of antifungal compounds. Besides this, yeast has been used to study antifungals against more evolutionary distant fungi (e.g., filamentous fungi). The majority of the currently used antifungal drugs are active against *S. cerevisiae* making it a suitable model for both drug development and the elucidation of the mechanisms underlying drug’s action [15]. In this work we show that C816 is a fungicidal and induces apoptosis in *S. cerevisiae* making it a potential candidate to be used as an antifungal or as a lead molecule for antifungal development.

## 3. Experimental Section

### 3.1. Molecules Tested

Crambescidin-816, -800 and -830 (Figure 1) were purified from the sponge *Crambe crambe* as described in [38]. The compounds were dissolved in DMSO and were 95% pure (HPLC-MS). The final DMSO concentration for cell treatment was always lower than 0.2%. Control cultures were treated with the highest DMSO concentration used in each experiment, to rule out any solvent interference in the results observed.

### 3.2. Cell Strain and Culture

The *Saccharomyces cerevisiae* strain NCPF3191 (ECACC) was used for all the experiments reported in this work. Cells were grown in YPD (20 g/L bacteriological peptone, 10 g/L yeast extract, 20 g/L glucose; SIGMA) at 30 °C with agitation. For culture maintenance, cells grown on YPD-agar (20 g/L bacteriological peptone, 10 g/L yeast extract, 20 g/L glucose, 15 g/L agar; SIGMA) were regularly re-plated.

### 3.3. Cell Growth Inhibition Determination

Growth inhibition of *S. cerevisiae* in the presence of crambescidins was determined by a spectrophotometric bioassay to determine the MIC_50_ (the antimicrobial concentration the inhibits 50% of the growth) based on the change of absorbance at 600 nm of yeast cultures in 48 well plates. An overnight culture was diluted to a concentration of 3 × 10^7^ cells per mL as determined with a hematocymeter. After dilution, 3 × 10^5^ cells per well were seeded in 48 well plates and then treated with Crambescidin-816, -800 of -830. The plates were incubated at 30 °C in a shaking incubator for 24 h. Absorbance was determined prior to treatment and up to 24 h of incubation with shacking at 30 °C, in a SYNERGY 4 spectrophometer (BIO-TEK).

### 3.4. Determination of Fungicidal Effect

The fungicidal effect of C816 was determined as previously described [39] with modifications. In brief, overnight cultures of *S. cerevisiae* were diluted to 1 × 10^6^ cell/mL in YPD media and incubated in 48 well plates in the presence of C816 or vehicle for 3 or 6 h at 30 °C with agitation. After incubation with the compound, cells were centrifuged and washed twice with YPD. Cells were resuspended in YPD media and cultured in 48 well plates up to 24 h at 30 °C with agitation. The optical density at 600 nm was determined as a measure of cell growth after the 24 h incubation elapsed.

To determine cell survival after C816 treatment we also performed serial dilutions of control and treated cultures and plated the cells on YPD-agar plates. Cells were incubated for 48 h at 30 °C and colonies were counted. Each treatment was assayed in triplicate to determine the percentage of cell survival in C816 treated cultures respect to controls.

### 3.5. Cell Cycle Assay by Flow Cytometry

An overnight culture of *S. cerevisiae* was diluted to 5 × 10^6^ cell per milliliter and incubated in 48 well plates in the presence of crambescidin-816 for 2, 4, 6, 10 and 24 h. After each incubation, cells were washed twice with PBS and then fixed with 70% ice cold ethanol for 1h at 4 °C. Fixed cells were washed twice with PBS and then incubated with 10 µg/mL propidium iodide (PI, SIGMA) and 0.1 mg/mL RNAse (SIGMA) dissolved in PBS for 1 h. Stained cells were sonicated for 1 min and 10,000 events for each sample were acquired with an AMNIS imaging flow cytometer using the AMNIS INSPIRE™ software. Data analysis was performed with the AMNIS IDEAS™ software. The experiments were performed by triplicate.

### 3.6. Determination of Apoptosis by Annexin V and Propidium Iodide (PI) Staining

To perform the assay, an overnight culture of *S. cerevisiae* was diluted to 5 × 10^6^ cell per milliliter and incubated in 48 well plates in the presence of crambescidin-816 for 10 or 24 h. After treatment, Annexin V staining was performed as previously described [40]. In brief, cells were washed with sorbitol wash buffer (1.2 M sorbitol, 0.5 mM MgCl_2_, 35 mM potassium phosphate, pH 6.8) and then digested with 20 U/mL of lyticase dissolved in sorbitol wash buffer for 1 h at 28 °C. Cells were then washed with sorbitol binding buffer (10 mM HEPES, 140 mM NaCl, 2.5 mM CaCl_2_, pH 7.4). The cells were resuspended in 40 µL of sorbitol binding buffer and two microliters of Annexin V (IMMUNOSTEP) and 1 µL of 500 µg/mL PI were added. Cells were stained for 20 min at room temperature in the dark and then washed in sorbitol binding buffer. Finally the cells were resuspended in sorbitol binding buffer and observed under a fluorescence microscope, or analyzed by flow cytometry.

### 3.7. Determination of Mitochondrial Transmembrane Potential (ΔΨ_m_)

Changes in mitochondrial ΔΨ_m_ were determined as previously described [41]. Control and C816 treated cells were washed with PBS and then resuspended in PBS with 2 µg/mL JC-1 (5,5′,6,6′-tetrachloro-1,1′,3,3′-tetraethylbenzimidazolylcarbocyanine iodide) and 2% glucose. Cells were stained for 30 min at 37 °C in the dark. After staining, cells were washed with PBS, resuspended in PBS supplemented with 2% glucose and analyzed by imaging flow cytometry. To perform compensation and as a positive control for mitochondrial uncoupling, FCCP (carbonyl cyanide 4-(trifluoromethoxy) phenylhydrazone) treated *S. cerevisiae* were used. This compound is a protonophore that uncouples oxidation from phosphorylation in mitochondria. FCCP, 10 µM final concentration, was added at the same time as JC-1. Ten thousand events were acquired of each sample detecting fluorescence in the green and orange channels. The relative mitochondrial fluorescence (RMF) was defined as the ratio aggregated JC-1 (orange):Monomeric JC-1 (green), which reflects the changes in ΔΨ_m_ per unit of mitochondrial mass.

### 3.8. Statistics

The results were analyzed using the SIGMAPLOT^®^ software. One way ANOVA was used for comparison of differences among groups. The Holm-Sidak multiple-range test was used for comparisons of differences between groups. A *p* < 0.05 was considered significant.

## 4. Conclusion

In conclusion, we show that C816 has a fungicidal effect in yeast and is more potent than C830 and the previously postulated lead for antifungal development C800. *S. cerevisiae* in the presence of C816 increases its size as a consequence of higher DNA content. The most potent compound tested induced mitochondrial dysfunction and apoptosis in yeast as determined by the appearance of a sub-G0/G1 population, phosphatidylserine translocation and collapse of the mitochondrial ΔΨ_m_ in treated cells. This work indicates that C816 could be a good lead for antifungal development.

## Supplementary Files

Supplementary File 1 Supplementary Information (PDF, 287 KB)
