# Supplementary material for: Crambescidin-816 Acts as a Fungicidal with More Potency than Crambescidin-800 and -830, Inducing Cell Cycle Arrest, Increased Cell Size and Apoptosis in Saccharomyces cerevisiae
Source: Mar Drugs. 2013 Nov 8;11(11):4419–34. doi: 10.3390/md11114419 (PMC3853736; doi:10.3390/md11114419)

Supplementary Information

Figure S1. (**a**) Percentage of live or necrotic cells after *S. cerevisiae* treatment with C816 or vehicle for 10 h. No increase of permeabilized cells was detected after C816 treatment. Ethanol permeabilized *S. cerevisiae* were used as positive control for PI nuclei staining. (**b**) Fluorescence microscopy analysis of C816 treated *S. cerevisiae* as in **a**. Merged PI staining and bright field for each treatment are shown.

Figure S2. (**a**) Histograms obtained after *S. cerevisiae* treatment with C816 or vehicle for 24 h, showing an increase in the subC1 population in 700 and 1000 nM treated cells. (**b**) Selected images of subC1 cells obtained by imaging flow cytometry. Ch1: Side scatter, Ch2: Bright field, Ch5: PI stained DNA, Ch2-Ch5: Composite obtained merging Ch2 and 5.

Figure S3. Representative images for healthy (control cells), Annexin V and Annexin V-PI stained cells (C816 treated cells).

Figure S1. (**a**) Percentage of live or necrotic cells after *S. cerevisiae* treatment with C816 or vehicle for 10 h. No increase of permeabilized cells was detected after C816 treatment. Ethanol permeabilized *S. cerevisiae* were used as positive control for PI nuclei staining. (**b**) Fluorescence microscopy analysis of C816 treated *S. cerevisiae* as in **a**. Merged PI staining and bright field for each treatment are shown.

a

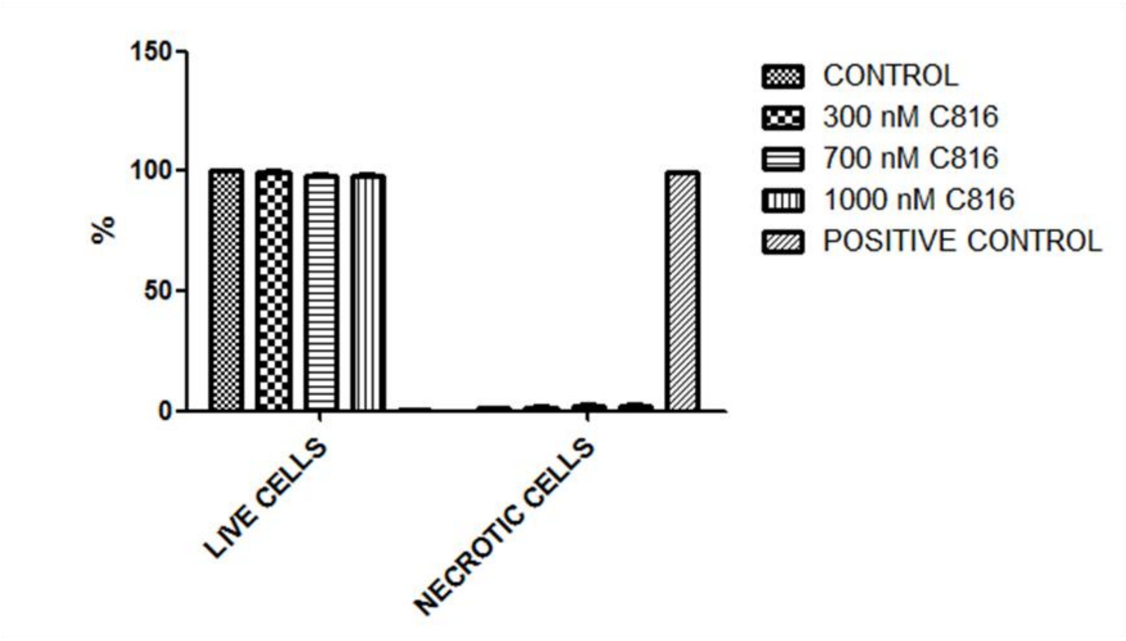

b

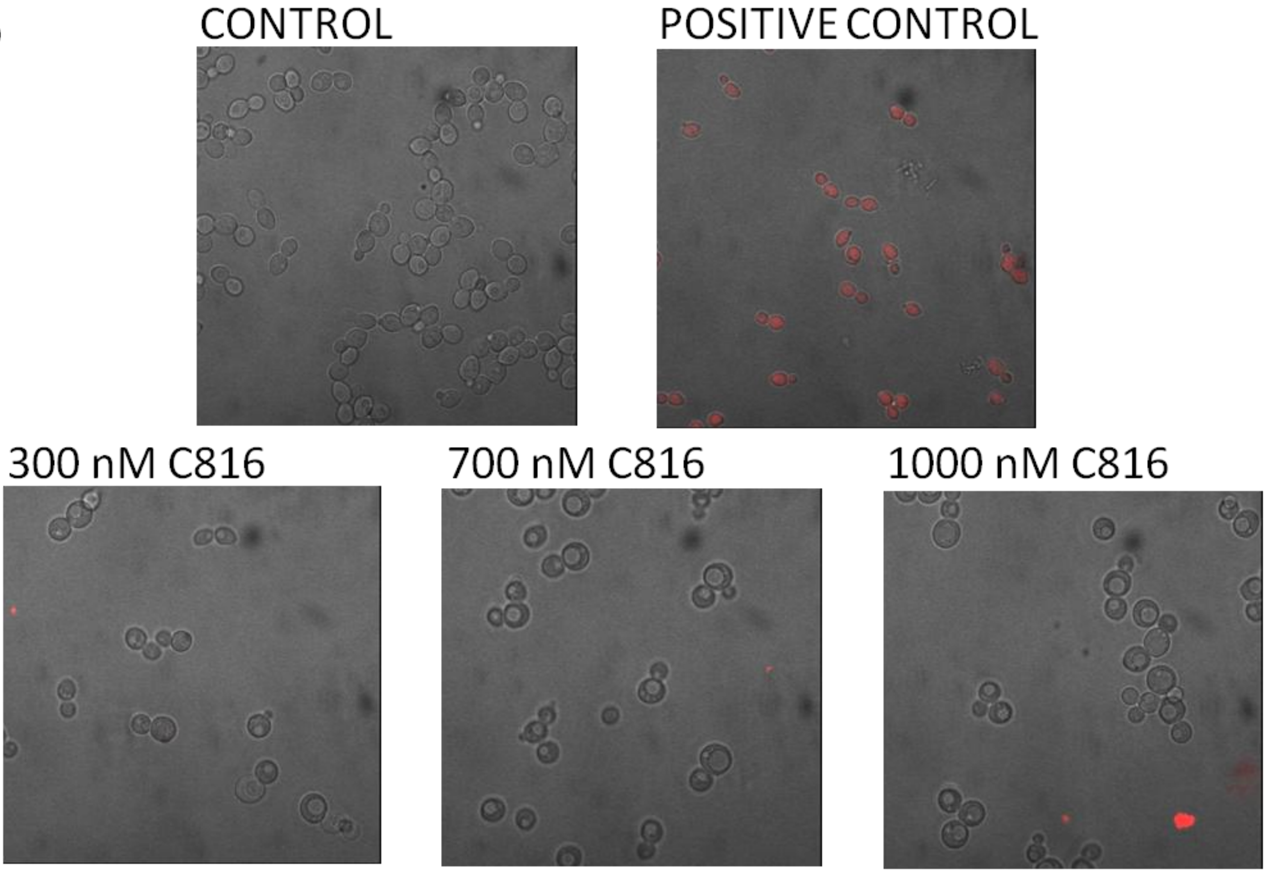

Figure S2. (**a**) Histograms obtained after *S. cerevisiae* treatment with C816 or vehicle for 24 h, showing an increase in the subC1 population in 700 and 1000 nM treated cells. (**b**) Selected images of subC1 cells obtained by imaging flow cytometry. Ch1: Side scatter, Ch2: Bright field, Ch5: PI stained DNA, Ch2-Ch5: Composite obtained merging Ch2 and 5.

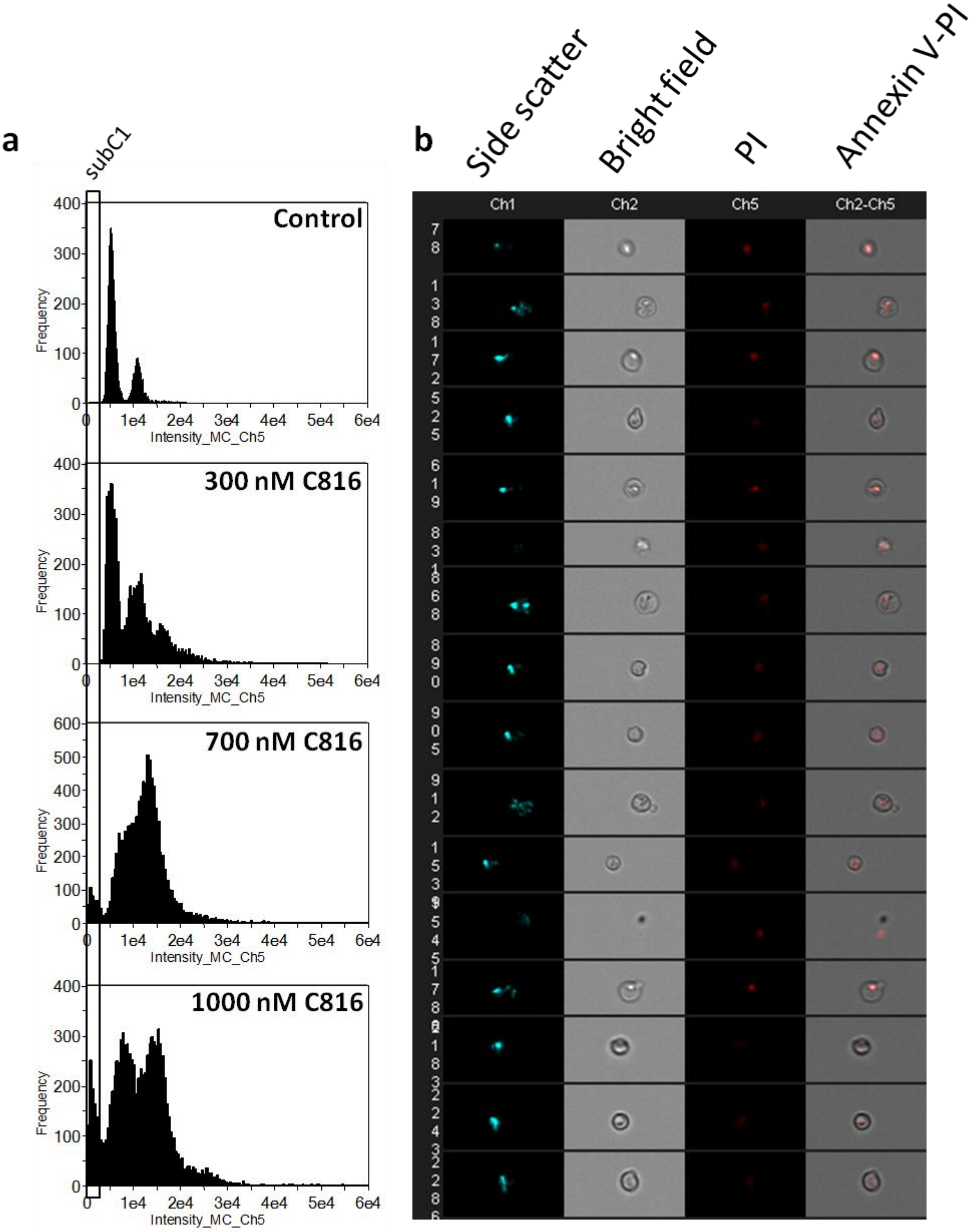

Figure S3. Representative images for healthy (control cells), Annexin V and Annexin V-PI stained cells (C816 treated cells).

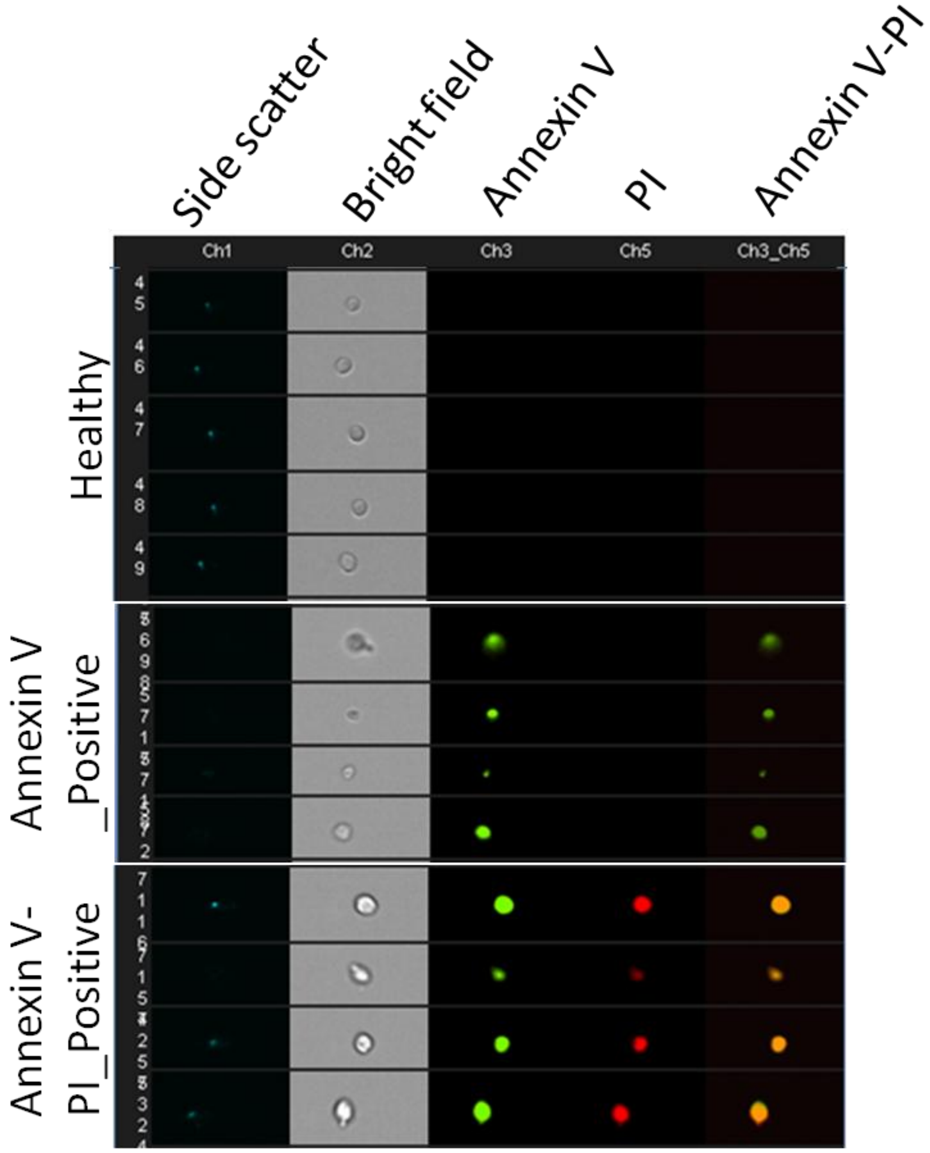

Supplement: Supplementary File 1 — Supplementary Information (PDF, 287 KB) [file marinedrugs-11-04419-s001.pdf]
